# Calmodulin-Cork Model of Gap Junction Channel Gating—One Molecule, Two Mechanisms

**DOI:** 10.3390/ijms21144938

**Published:** 2020-07-13

**Authors:** Camillo Peracchia

**Affiliations:** Department of Pharmacology and Physiology, School of Medicine and Dentistry, University Rochester, Rochester, NY 14642, USA; camillo.peracchia@gmail.com

**Keywords:** gap junctions, connexins, channel gating, calcium, calmodulin, cell communication, cell-to-cell channels, cell coupling, cell uncoupling

## Abstract

The Calmodulin-Cork gating model is based on evidence for the direct role of calmodulin (CaM) in channel gating. Indeed, chemical gating of cell-to-cell channels is sensitive to nanomolar cytosolic calcium concentrations [Ca^2+^]_i_. Calmodulin inhibitors and inhibition of CaM expression prevent chemical gating. CaMCC, a CaM mutant with higher Ca^2+^-sensitivity greatly increases chemical gating sensitivity (in CaMCC the NH_2_-terminal EF-hand pair (res. 9–76) is replaced by the COOH-terminal pair (res. 82–148). Calmodulin colocalizes with connexins. Connexins have high-affinity CaM binding sites. Several connexin mutants paired to wild-type connexins have a high gating sensitivity that is eliminated by inhibition of CaM expression. Repeated transjunctional voltage (Vj) pulses slowly and progressively close a large number of channels by the chemical/slow gate (CaM lobe). At the single-channel level, the chemical/slow gate closes and opens slowly with on-off fluctuations. The model proposes two types of CaM-driven gating: “Ca-CaM-Cork” and “CaM-Cork”. In the first, gating involves Ca^2+^-induced CaM-activation. In the second, gating takes place without [Ca^2+^]_i_ rise. The Ca-CaM-Cork gating is only reversed by a return of [Ca^2+^]_i_ to resting values, while the CaM-Cork gating is reversed by Vj positive at the gated side.

## 1. Introduction

In most tissues, cells in contact with each other exchange cytosolic molecules of low molecular weight via channels aggregated at gap junctions. Gap junction mediated cell-to-cell communication allows neighboring cells to coordinate and regulate many functional activities in mature and developing organs [1,2,3]. A gap junction channel is made of the interaction of two hemichannels (connexons/innexons) that form a hydrophilic pathway across the two apposed plasma membranes and the extracellular space (gap). Each connexon/innexon is an oligomer of six proteins (connexins/innexins) that span the plasma membrane and create a hydrophilic pore insulated from lipid bilayer and extracellular medium.

Gap junction channels have been thought to possess as many as four types of gates: fast transjunctional voltage (Vj) gate, slow Vj-gate, chemical gate and gate sensitive to membrane potential (Vm). However, since the behavior of the slow Vj-gate and the Vm-sensitive is the same as that of the chemical gate, most likely these gates are the same. We have named this gate “chemical/slow gate” [1].

In 2000, we proposed a calmodulin (CaM)-mediated “cork-type” gating model [4]. The model proposes two mechanisms. One, “Ca-CaM-Cork”, envisions physical blockage of the channel’s mouth by a CaM lobe (N-lobe?), likely to be combined with conformational connexin changes induced by Ca^2+^-CaM binding to connexin sites. The other, “CaM-Cork”, also proposes a physical blockage of the channel’s mouth by a CaM lobe, but without Ca^2+^-activation. The first is only reversed by the return of intracellular Ca^2+^ concentration ([Ca^2+^]_i_) to resting values. The latter is reversed by Vj positive at the gated side.

## 2. Why Calmodulin?

### 2.1. Gating is Activated by Nanomolar Calcium Concentrations [Ca^2+^]_i_

Numerous studies have demonstrated that [Ca^2+^]_i_ in the high nanomolar range are effective on channel gating in many vertebrate and invertebrate cells. They include: cardiomyocytes [5,6,7], crayfish axons [8,9], *Xenopus laevis* oocytes [10], rat lacrimal epithelial cells [11], Novikoff hepatoma cells [12,13], astrocytes [14,15,16], lens-cultured cells [17], human fibroblasts [18], cultured cells expressing Cx43 [19], pancreatic cells [20,21,22,23,24,25] and neuro-2a cells (N2a) expressing Cx43, among others.

Much of our work on chemical gating has tested cell uncoupling resulting from cytosolic acidification. Since low pH_i_ had been reported to cause chemical gating by acting directly on the channel’s gates [26], staring in 1990 we questioned extensively whether the H^+^_i_ effect is direct or mediated by an increase in [Ca^2+^]_i_. Our data, obtained in a variety of vertebrate [10,12] and invertebrate [8,9,27] cells, have convinced us that cytosolic acidification does not directly affect the channel gates, but rather its effect is mediated by Ca^2+^_i_; rev. in [1,28]. In fact, most relevant in this respect are our findings in Novikoff hepatoma cells, in which pH_i_ = 6.1 does not affect gating as long as [Ca^2+^]_i_ is buffered to low nanomolar concentrations by 1,2-bis(*o*-aminophenoxy) ethane-*N,N,N^1^,N^1^*-tetraacetic acid) (BAPTA) added to the patch–pipette solutions [12].

Based on gating sensitivity to nanomolar [Ca^2+^]_i_, it is reasonable to believe not only that Ca^2+^_i_ is a fine modulator of cell-to-cell communication, but also that a Ca^2+^- modulated protein, CaM being the most likely, mediates the Ca^2+^_i_ -induced channel gating. Direct action of Ca^2+^_i_ is very unlikely because the cytosolic domains of connexins do not contain high-affinity Ca^2+^-binding sites. In contrast, they do have high-affinity CaM binding sites [1] (see in the following).

### 2.2. CaM Inhibitors Prevent Chemical Gating

Several decades ago, we first proposed the role of CaM in gap junction channel gating based on evidence that CaM inhibitors prevent chemical uncoupling of *Xenopus* embryonic cells [29,30,31]. Over the years, various CaM inhibitors have been reported to be effective in inhibiting chemical gating. They include: trifluoperazine (TFP) [30,31], calmidazolium (CDZ) [19,29,32] and N-(6-aminohexyl)-5-chloro-1-naphthalenesulfonamide hydrochloride (W7) [32,33,34,35,36].

### 2.3. Inhibition of CaM Expression Prevents Chemical Gating

Inhibition of CaM expression by injection of oligonucleotides antisense to the two CaM mRNAs expressed in oocytes results in progressive loss of uncoupling efficiency in oocytes expressing the native connexin Cx38 [10], Cx32-mutants [37] or Cx45 channels [38]. Chemical gating efficiency partially recovers with CaM injection [10].

### 2.4. A CaM Mutant with Higher Ca^2+^-Sensitivity Greatly Enhances Gating Sensitivity

**Figure 1:**
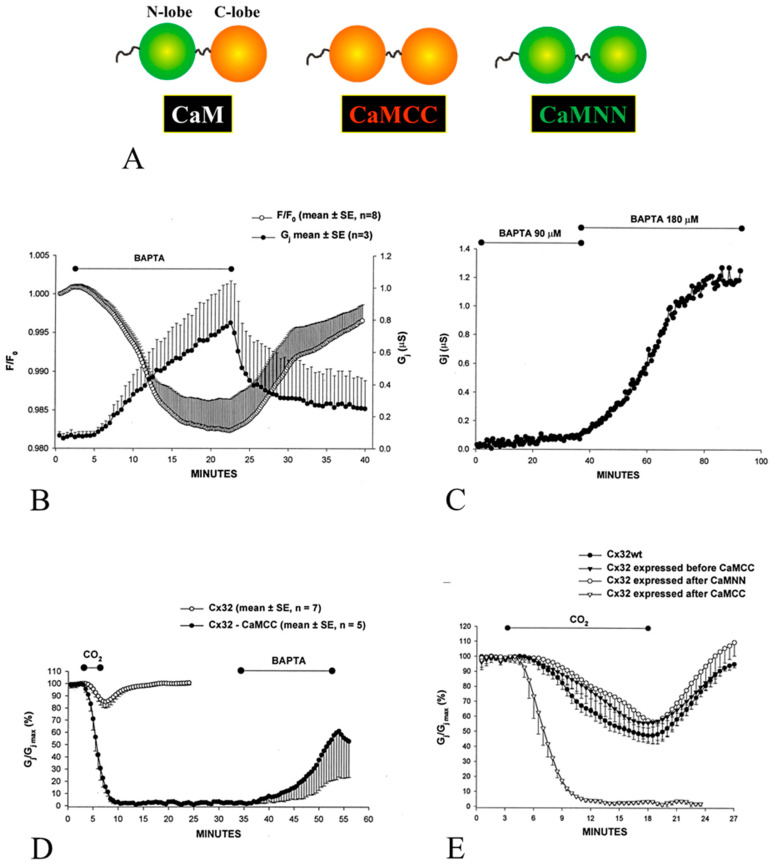
In oocytes expressing CaMCC (**A**) before Cx32, Gj is very low (**B**), but rapidly increases with 180 µM BAPTA superfusion (**B**,**C**) as [Ca^2+^]_i_, measured with Calcium Green-1, drops (**B**, F/F0). sensitivity. In CaMCC the NH_2_-terminal EF-hand pair (res. 9–76) is replaced by the COOH-terminal pair (res. 82–148). Lower [BAPTA] (90 µM) are much less effective (**C**). Cx32 channels expressed after CaMCC are more sensitive to 100% CO_2_ than controls (**D**,**E**), as Gj rapidly drops to zero (**D**,**E**) with CO_2_ applications for as short as 3 min (**D**) or 15 min (**E**; mean ± standard error, SE, *n* = 3), while in controls it decreases by only ~15% even with CO_2_ applications as long as 15 min (E; mean ± SE, *n* = 7). After CO_2_ washout, Gj/Gjmax remains at 0% but rapidly increases with 180 µM BAPTA superfusion (**D**). Expression of Cx32 before CaMCC (**E**; mean ± SE, *n* = 3) or expression of CaMNN (A) has no effect on gating (**E**; mean ± SE, *n* = 3). From [39].

In 2000, we tested the CaM role in gating in *Xenopus* oocytes overexpressing the CaM mutant CaMCC (Figure 1) [39,40] by measuring junctional conductance (Gj). For measuring Gj, a transjunctional voltage (Vj) gradient is created by imposing a voltage pulse (V_1_) to one oocyte (oocyte 1) while maintaining V_2_ at membrane potential (Vm); thus, Vj = V_1_. The negative-feedback current (I_2_), injected by the clamp amplifier in oocyte 2 for maintaining V_2_ constant at Vm, is used for calculating Gj, as it is identical in magnitude to the junctional current (Ij), but of opposite sign (Ij = −I_2_); Gj = Ij/Vj (Ohm’s law). In CaMCC, the CaM’s NH_2_-terminal EF-hand pair (res. 9–76) is replaced by the COOH-terminal pair (res. 82–148; Figure 1A). Since the Ca^2+^-affinity of the carboxy-terminal EF-hand pair is greater than that of the NH_2_-terminal pair by approximately one order of magnitude [41], we thought that CaMCC expression might increase the sensitivity of chemical gating.

In fact, in oocytes expressing CaMCC and Cx32 Gj was minimal, but substantially increased when [Ca^2+^]_i_, monitored with Calcium Green-1, was lowered by 180 µM BAPTA superfusion (Figure 1B,C). Indeed, CaMCC enhances Ca^2+^-gating-sensitivity so much that gating is even activated by resting [Ca^2+^]_i_. Significantly, the effect of CaMCC is only observed when its expression precedes that of Cx32, which indicates that it successfully competes with CaM wild-type for Cx32 interaction. In contrast, the mutant CaMNN, in which the NH_2_-terminal pair replaces the COOH-terminal pair had no effect (Figure 1E), suggesting that CaMNN does not effectively compete against wild-type CaM for Cx32 interaction. The significance of the CaMCC/Cx32 sequence of expression indicates that CaMCC, as native CaM, interacts with Cx32 before connexon assembly.

The enhanced gating sensitivity induced by CaMCC’s expression was further confirmed by testing the effect of 100% carbon dioxide (CO_2_), which causes a drop in pHi to ~6.3 and results in an increase in [Ca^2+^]_i_. In the presence of CO_2_, Gj rapidly dropped to zero, while in controls it only decreased by ~15% (Figure 1D). With CO_2_ washout, Gj remained at zero indefinitely, but begun recovering (reversibly) with 180 µM BAPTA superfusion (Figure 1D), which obviously decreased [Ca^2+^]_i_ below resting values.

**Figure 2:**
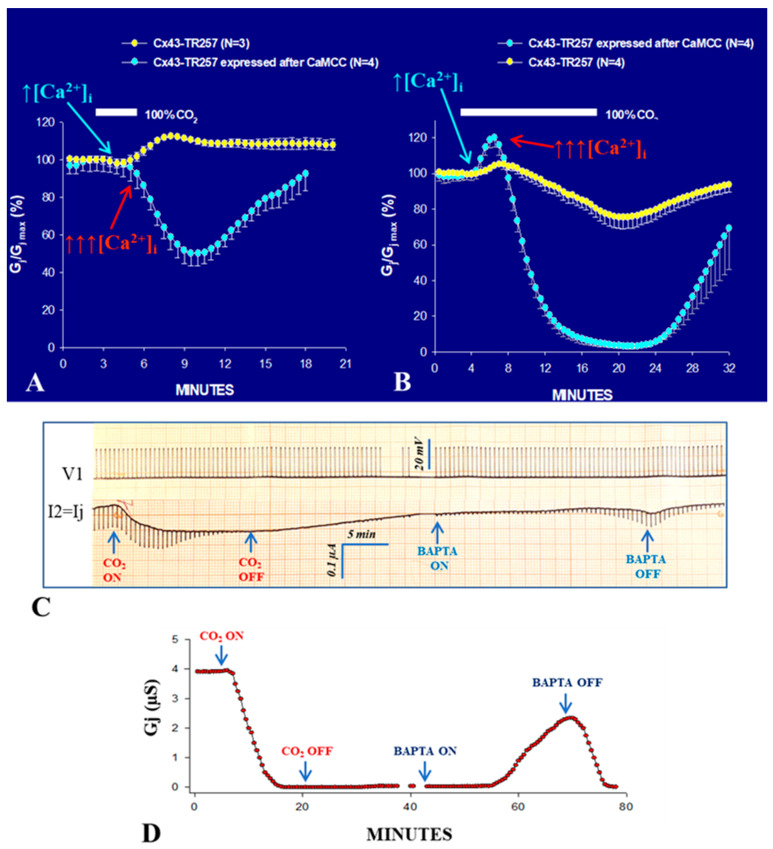
CT-deleted Cx43 channels (Cx43TR257), expressed after CaMCC, are much more sensitive to CO_2_ than controls (A and B), as Gj rapidly drops to ~50% (**A**) and nearly 0% (**B**) with 3 and 15 min 100% CO_2_ applications, respectively, while in controls Gj decreases by only ~25% even with 15 min CO_2_ (**B**). Curiously, in controls paradoxically Gj increases reversibly with brief applications of CO_2_ (**A**); similarly, Gj increases before dropping in control Cx43TR257 (**B**). The initial Gj rise may result from activation of the CaMKII cascade (see text). After CO_2_-washout, in some experiments, Gj remains indefinitely at nearly 0% (**C**,**D**), but reversibly increases with 180 µM BAPTA superfusion (**C**,**D**). An original voltage (V1 = Vj) and current (I2 = Ij) chart record is shown in C. The changes in Gj (µS) of the experiment shown in C are shown in D (Gj = Ij/Vj). These Cx43 data are mine, new and not published elsewhere.

The same result was obtained in oocytes expressing Cx43 or COOH-terminus (CT) deleted Cx43 (Cx43-TR257) after CaMCC expression (Figure 2)—note that these Cx43 data are new and not published elsewhere. Note that CT-deleted Cx43 (Cx43-TR257) has been reported to be relatively insensitive to 100% CO_2_ [42,43,44], which prompted the creation of the ball-and-chain model, which envisions a particle-receptor interaction in which the CT domain acts as a gating particle by binding to a separate region of the connexin. [43]. In contrast, Figure 2A,B demonstrates that Cx43′s CT domain is not needed for chemical gating, as also reported by Wei and coworkers [45]. As with Cx32 channels (Figure 1D), in some experiments, Cx43-TR257 channels expressed after CaMCC remained indefinitely uncoupled following CO_2_ washout (Figure 2C,D), but Gj reversibly recovered with 180 µM BAPTA (Figure 2C,D) as it did with Cx32 channels (Figure 1D).

Two phenomena are notable with Cx43-TR257 in the presence and absence of CaMCC:

1. With 3 min CO_2_ application, in Cx43-TR257 channels, the Gj time-course is monophasic, as paradoxically Gj just increases (Figure 2A). In contrast, with CaMCC the Gj change is biphasic, as Gj drops to ~50% following a brief rise (Figure 2A).
2. With 15 min CO_2_ application, in Cx43-TR257 channels, the Gj time-course is biphasic both in the presence and absence of CaMCC, as it rises before dropping (Figure 2B).

**Figure 3:**
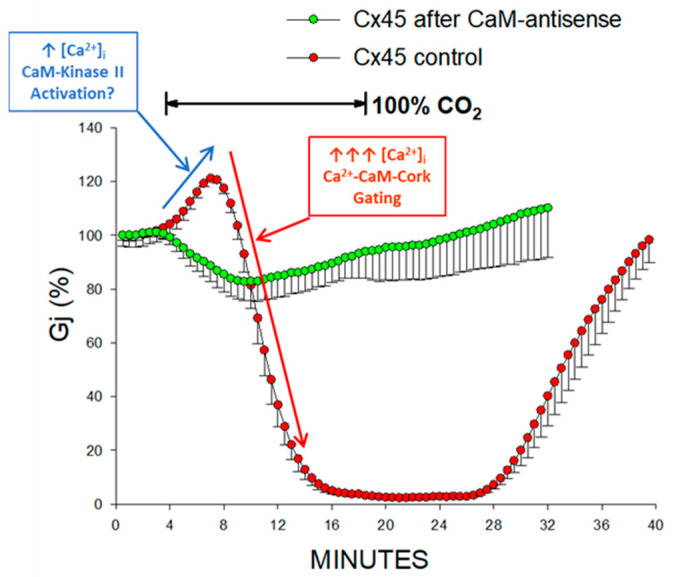
The junctional conductance (Gj), monitored in *Xenopus* oocyte pair expressing Cx45 during CO_2_ application, has a biphasic course: initial rise followed by a rapid drop to full uncoupling (red circles). Since activation of CaMKII increases Gj (see text), the initial Gj rise may result from activation of the CaMKII cascade and opening of dormant, CaM-Cork gated channels. We believe that subsequent rapid Gj drop results from the activation of the Ca-CaM-Cork mechanism. Inhibition of CaM expression greatly reduces the CO_2_ sensitivity, as Gj reversibly drops (monophasically) by only ~17% (green circles)—the absence of Gj rise suggests that with reducing [CaM]_i_ at rest, most channels are open. From [38].

Significantly, this biphasic time-course (Figure 2B) is also observed often in different wild-type connexin channels. An example is provided by Cx45 channels (Figure 3) [38]. We have suggested that the early Gj rise may result from activation of the Ca/CaM-Kinase II (CaMKII) cascade [38], which is known to increase Gj [46,47] and open Cx43 hemichannels [48]. If this were the case, a likely scenario could be that with CO_2_ application an early, moderate, [Ca^2+^]_i_ rise (↑[Ca^2+^]_i_; Figure 2A,B and Figure 3) opens a number of dormant channels, likely to be in closed state via the CaM-Cork mechanism (see in the following) (Figure 2A,B, and Figure 3), perhaps via activation of the CaMKII cascade. In contrast, the subsequent, larger [Ca^2+^]_i_ rise (↑↑↑[Ca^2+^]_i_; Figure 2A,B and Figure 3) would close the channels by direct Ca^2+^-CaM interaction (Ca-CaM-Cork gating; see in the following) (Figure 2 and Figure 3). The reason why we believe that the early Gj rise of Cx43 channels results from moderate [Ca^2+^]_i_ rise is that with brief (3 min) exposure to CO_2_ the Gj curve is just monophasic (Figure 2A; yellow circles). This may suggest that a moderate [Ca^2+^]_i_ may activate the CaMKII cascade, but is not enough to activate the Ca-CaM-Cork gating mechanism. In our Ca-Cork model, the gating element is suggested to be the CaM’s N-lobe, which has a lower Ca-sensitivity than the C-lobe. This could be the reason why the CaMKII cascade is activated a lower [Ca^2+^]_i_ that the CaM’s N-lobe.

Evidence that both phenomena are eliminated by inhibition of CaM expression (Figure 3) confirms the CaM-participation in this phenomenon. Although there is no evidence that CaMKII directly phosphorylates Cx45 channels, it is known that it phosphorylates Cx43′CT serine residues [49]. While in Cx43TR257 most of the serine residues of CT [49] are lost, three of them (S244, S255 and S257) are preserved. The loss of CO_2_-induced Gj rise with inhibition of CaM expression suggests that with CaM inhibition, unlike in controls, most of the channels are in an open state. It is likely that the reduced CaM concentration prevents some channels from being closed by the CaM-Cork gating mechanism at rest.

### 2.5. Colocalization of CaM and Connexins

**Figure 4:**
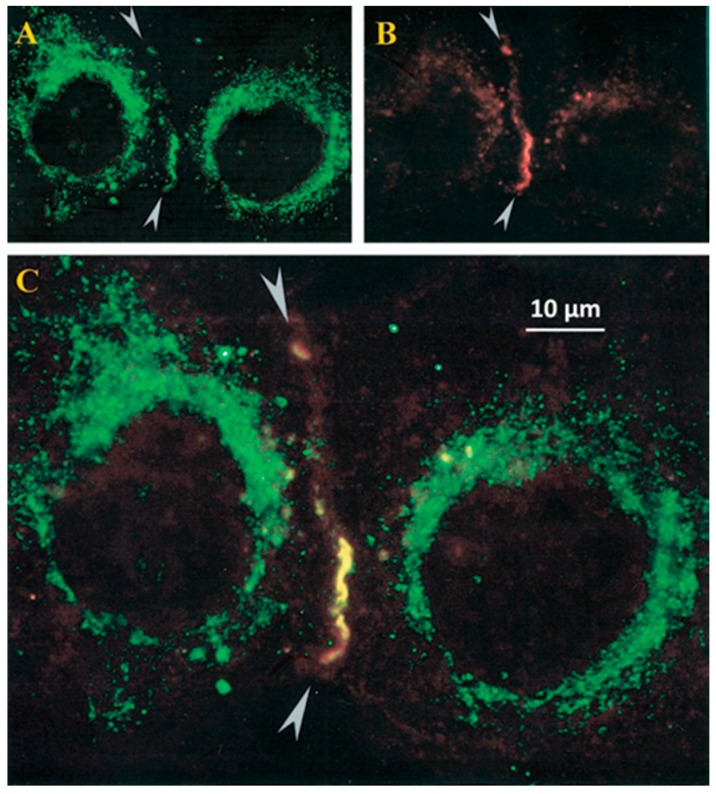
Immunofluorescence labeling of CaM (**A**) and Cx32 (**B**) in HeLa cells expressing Cx32. CaM and Cx32 colocalize in linear (regions between arrowheads) and punctated areas of cell–cell contact (**C**). Labeling is also seen in the cytoplasm (**A**–**C**); this is likely to correspond to CaM and CaM linked to Cx32 in cytoplasmic organelles. Scale bar 10 μm. From [39].

Calmodulin–connexin interaction was tested by immunofluorescence microscopy [39,40]. In HeLa cells expressing Cx32, CaM and Cx32 colocalize at cell–cell contact as well as in few cytoplasmic spots (Figure 4) [39,40]. Similar data were obtained with Cx43 and Cx37 (Sotkis and Peracchia, unpublished data), and with Cx50 [50,51] and Cx36 [52]. 

The CaM-Cx32 colocalization was further proven by confocal fluorescence microscopy in HeLa cells expressing Cx32 linked to the green fluorescent protein (Cx32-GFP) and CaM linked to the red fluorescent protein (CaM-RFP) [1,40,53]. In these cells, however, CaM and Cx32 only colocalized in the cytoplasm, as these cells did not form gap junctions, probably because steric hindrance prevented connexin oligomerization into connexons [40,53].

**Figure 5:**
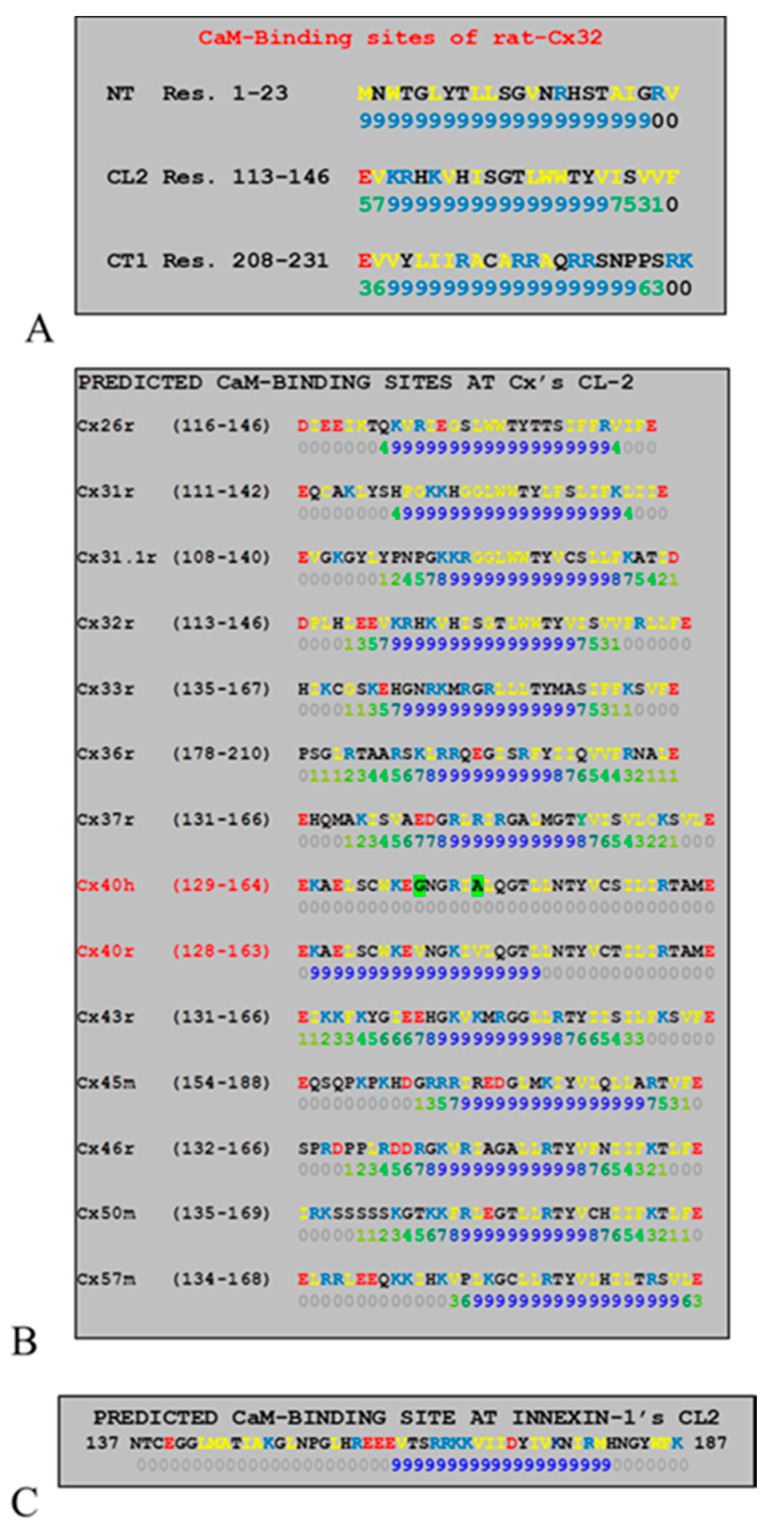
Most connexins have three CaM-binding sites. The CaM-binding sites of rat-Cx32: NH_2_-terminus (NT), second half of the cytoplasmic loop (CL2) and initial domain of the COOH-terminus (CT1) are shown in (**A**). Most relevant for chemical gating is likely to be the CL2 site of connexins (**B**) and Innexin-1 (**C**). The CaM-binding site was identified by a computer program that rate the sites 0–9 [73].

Recently, direct visualization of CaM-Cx45 interaction was reported in live cells by Bioluminescence Resonance Energy Transfer (BRET) [54]; the CaM-Cx45 interaction was Ca^2+^-dependent and was prevented by W7. The interaction involved the CaM-binding site in the second half of the cytoplasmic loop (CL2, res. 164–186; Figure 5). This was confirmed by the high-affinity binding of fluorescence-labeled CaM to a peptide matching the CL2 domain [54]. In fact, there is evidence for Ca^2+^-dependent and independent CaM-binding to the CL2 sequence of Cx45 [55,56]. The Ca^2+^-independent CaM-CL2 binding confirms earlier evidence that CaM is linked to connexins at resting [Ca^2+^]_i_ (~50 nM) [37,39,40,53,54,56].

### 2.6. Connexins Have High-Affinity CaM Binding Sites

Calmodulin binding sites were first identified in 1988 in Cx32: one at NH_2_-terminus (NT) and one at the initial domain of the COOH-terminus (CT1) [57] (Figure 5A). Their binding to CaM was proven by several studies [58,59,60]. CaM also binds to CT1 of Cx43 [61], Cx35 and Cx34.7 [52,62] and Cx36 [52]. However, the CT1 site is unlikely to be relevant for chemical gating because neither Cx32′s CT deletion by 84% [63,64] nor Cx43′s CT deletion at res. 257 affects chemical gating efficiency [45]. In contrast, CL2 (Figure 5A) is likely to be most relevant to chemical gating [65,66] (Figure 5B,C). 

**Figure 6:**
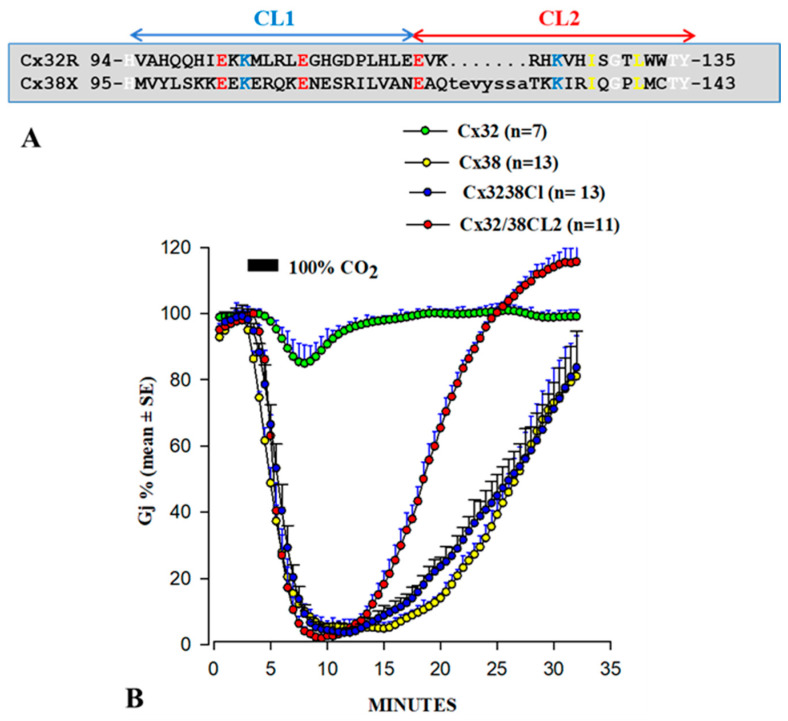
Sequences of the cytoplasmic loop (CL) of rat Cx32 and *Xenopus* Cx38 (**A**). Gj changes induced by 100% CO_2_, monitored in oocytes expressing Cx32, Cx38, or Cx32/38 chimeras are shown in B. Channels made of Cx32/38CL (Cx32′s CL being replaced by that of Cx38) or Cx32/38CL2 (Cx32′s CL2 being replaced by that of Cx38) reproduce precisely the gating efficiency of Cx38 channels in both magnitude and rate (**B**), but Gj recovers faster in Cx32/38CL2 channels. Note that CL2 contains a CaM-binding site (see Figure 5A,B). Adapted from [65,66].

We first focused on the CL2 domain in 1996, when we tested in oocytes the CO_2_ sensitivity of channels formed by Cx32 and Cx38 chimeras and mutants. As mentioned earlier, Cx32 and Cx38 make channels with opposite CO_2_ gating sensitivity (Figure 6B) [65,66]. Channels made of Cx32/38CL (Figure 6A; Cx38′s CL replaced by that of Cx32) reproduced almost perfectly Cx38 channel gating in magnitude and time course (Figure 6B) [66]. For identifying more precisely the most relevant CL domain, we expressed Cx32/Cx38 chimeras in which either the 1st half (CL1) or the 2nd half of Cx38′s CL replaced that of Cx32(CL2)(Figure 6A) [65]. Cx32/Cx38CL2 channels (Cx32 with Cx38′s CL2) were like Cx38 channels in terms of CO_2_ sensitivity, but Gj recovered faster (Figure 6B). Significantly, they matched Cx32 channels in fast-Vj gating sensitivity [65]. The data indicate that CL1 and CL2 contain domains relevant to fast-Vj- and chemical-gating, respectively [65]. This is consistent with evidence that CL2 contains a CaM binding site (Figure 5B,C) [1]. 

For testing the gating efficiency of channels made of Cx43 mutants that lack the CL2′s CaM-binding site, Zhou and coworkers expressed in HeLa cells two mutants linked to EYFP (a fluorescent protein) [67]. The lack of the site abolished Ca^2+^-dependent gating, confirming that CL2 contains the CaM-binding domain relevant to chemical gating [67]. The importance of this site was further confirmed with Cx43 [19], Cx50 [68], or Cx44 [69,70] channels.

A study tested by small-angle X-ray scattering a synthetic peptide matching the CL2′s CaM-binding domain of Cx43 (res. 144–158), in order to detect potential Ca^2+^-induced conformational changes [71]. With peptide interaction, CaM assumed a more globular conformation, suggesting that CaM binds to the peptide in typical “collapsed” conformation [71].

Xu and coworkers [19] reported that ionomycin application increases [Ca^2+^]_i_ and causes Gj to drop by 95% in N2a cells expressing human Cx43, but not in cells expressing human Cx40. This at first appeared to contradict our evidence for great gating sensitivity of rat Cx40 channels [72]. However, it might be consistent with the role of Ca^2+^-CaM in gating, because a computer analysis of CL2′s CaM binding sites indicates that the rat-Cx40 site is absent in human Cx40 (Figure 5B) [1]. The Ca^2+^-induced Gj drop in Cx43 channels was prevented by CDZ and reversed by adding EGTA to the Ca^2+^-free saline [19]. Significantly, the addition to patch–pipette solutions of a peptide that matched the CL2 CaM-binding site of Cx43 (res.136–158) also prevented gating. In contrast, neither a scrambled peptide nor the inhibitory peptide (res. 290–309) of CaMKII did so [19].

An analysis of CL2 sequences as potential CaM-binding domains in mammalian connexins and the invertebrate Innexin-1 by a program that rate the sites 0–9 [73], shows that in all connexins and Innexin-1, but in human Cx40, the CL2 sequence contains a potential CaM-binding site (Figure 5B,C). It should be stressed, however, that the high-affinity to this site may not directly correlate with the higher sensitivity of Ca-CaM gating. CaM–CL2 interaction was confirmed by Jenny Yang’s team for Cx43 [67], Cx44 [70] and Cx50 [68] and by Katalin Török’s team for Cx32, Cx35, Cx45 and Cx57 [55,56].

### 2.7. CaM Is Linked to Connexins at Resting [Ca^2+^]_i_

There are reasons to believe that CaM is linked to connexins at resting [Ca^2+^]_i_ as well. There is evidence that CaMCC overexpression greatly decreases the Vj sensitivity of Cx32 channels [39]. Furthermore, the Vj sensitivity of Cx45 channels is significantly inhibited by blocking CaM expression [38]—note that Cx45 channels are quite sensitive to Vj and the chemical/slow gate mediates Vj gating [74]. The behavior of mutant/Cx32 channels also indicates that CaM is linked to connexins at resting [Ca^2+^]_i_ (~50 nM), because inhibition of CaM expression in mutant/Cx32 channels greatly decreases the effectiveness of Vj on Gj [37]. Even more relevant is immunofluorescent evidence that CaM colocalizes with connexins at gap junctions of the cultured cell at resting [Ca^2+^]_i_ (Figure 4) [39,40].

Evidence that CaM is linked to connexins at resting [Ca^2+^]_i_ has been recently confirmed by an in vitro study that tested CaM-binding to peptides matching the CL2′s CaM binding site of Cx32, Cx35, Cx45 and Cx57, with and without Ca^2+^ [55,56]. Fluorescence changes of the double-labeled FRET (Föster Resonance Energy Transfer) probe and Ca^2+^-sensitive TA–CaM (2-chloro-(epsilon-amino-Lys75)-[6-[4-(N,N-diethylamino)phenyl]-1,3,5-triazin-4-yl]calmodulin) were revealed by fluorescence spectroscopy and stopped-flow fluorimetry [75] at physiological ionic strength (pH 7.5, 20 °C). The following kD values were obtained (Table 1):

### 2.8. Potential Role of CaM-Activated Enzymes in Chemical Gating?

The possibility that CaM induces channel gating via enzyme activation, was tested with inhibitors and/or activators of many enzymes [53]. However, none of them affected Gj and/or gating efficiency [53]. The potential role of Ca^2+^-proteases is also unlikely because proteolysis would be irreversible, while the recovery rate of Ca^2+^-induced uncoupling is much faster than the turnover-time of connexins’ (half-life = ~3 h) [76].

## 3. Why Cork?

### 3.1. Behavior of Heterotypic Mutant-Wildtype Channels

**Figure 7:**
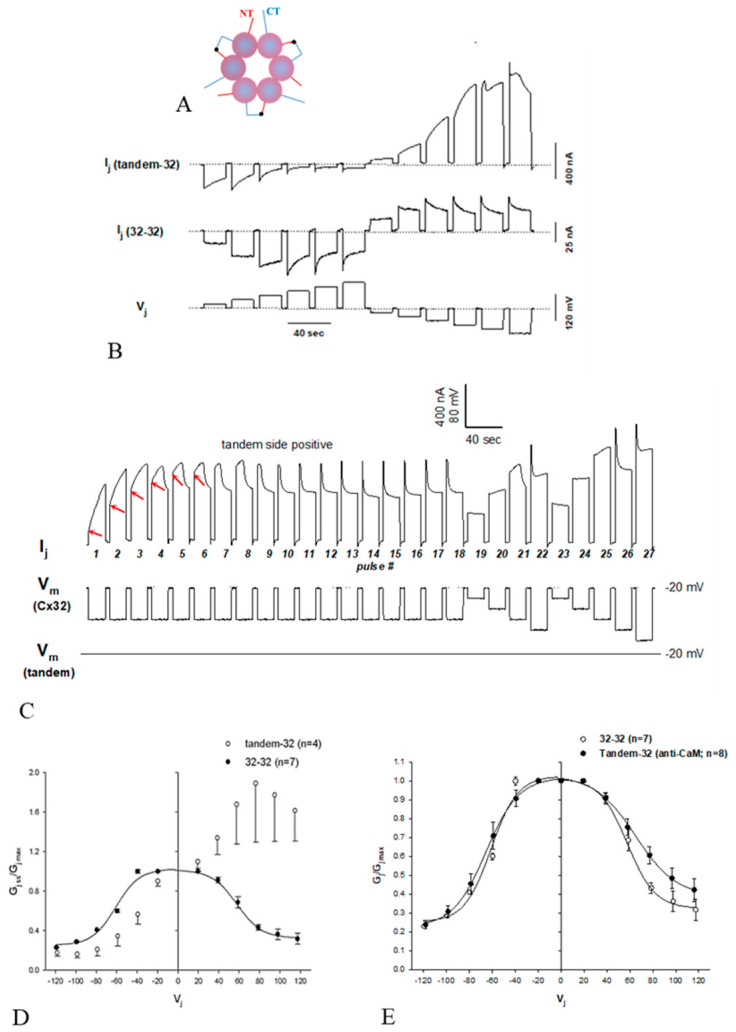
Ij/Vj records of *Xenopus* oocyte pairs expressing either homotypic Cx32 channels (32–32) or heterotypic tandem-32 channels (**A**,**B**). While 32–32 channels manifest a characteristic Ij sensitivity to Vj (**B**), tandem-32 channels show an unusual behavior (**B**). With mutant side negative (**B**, left), initial and final Ij gradually drop to very low values, while with mutant side positive (**B**, right), Ij gradually increases to high values and only at Vj = 100–120 mV, a more typical Ij behavior appears. With repeated application of 60-mV Vj pulses (tandem side positive), 3 types of Ij behavior are observed: monophasic Ij increase (**C**, pulses 1–3), biphasic Ij time-course (**C**, pulses 4–9 and conventional Ij behavior (**C**, pulses 10–18). Subsequent applications of the Vj protocol (tandem side positive) result in fairly normal Ij behaviors (**C**, pulses 19–27). The asymmetric Ij-Vj behavior of tandem-32 channels is demonstrated in plots of normalized Gj versus Vj (**D**). Significantly, the asymmetrical Ij/Vj behavior is not observed following inhibition of CaM expression (**E**). (**B**,**C**) from [77]; (**D**,**E**) from [37].

**Figure 8:**
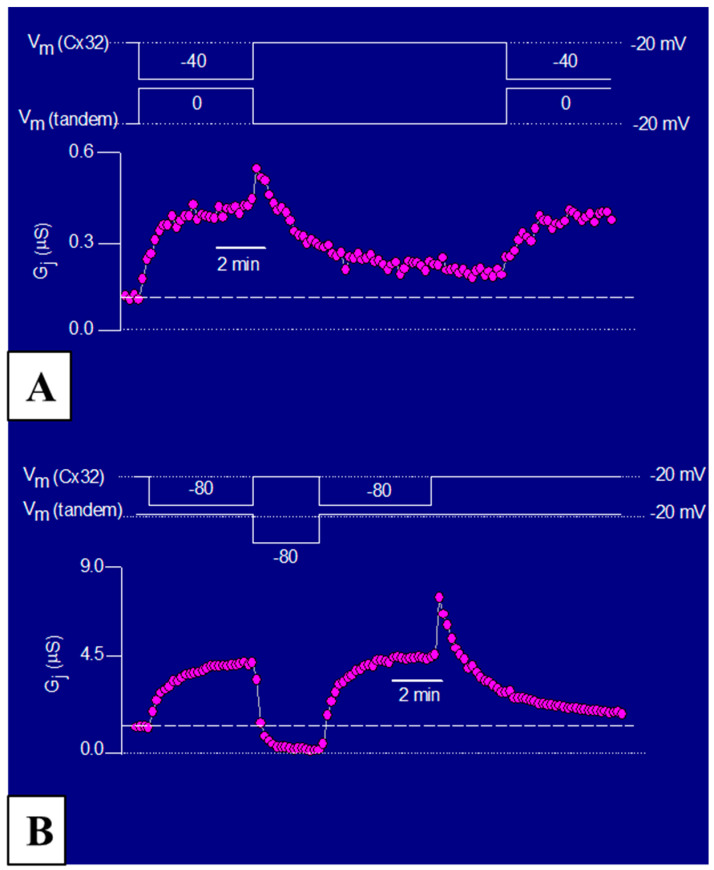
Response of Gj to steady-state Vj in tandem-32 channels (**A**,**B**). In oocytes initially clamped at Vm = 20 mV (Vj = 0), a 40–60 mV Vj step (tandem side positive) exponentially increases Gj in tandem-32 channels (**A**,**B**). Upon return to Vj = 0 mV from Vj = 40 mV (**A**), Gj decreases exponentially to near control values. With Vj reversal to 60 mV (tandem side negative), Gj decreases exponentially below control values (**B**), indicating that Vj negative at the tandem side actively closes channels. Upon return to Vj = 0 from Vj = 40 (**A**) or 60 (**B**) mV (tandem side positive), Gj increases abruptly before dropping (**A**,**B**), because the fast Vj-gates of the Cx32 side reopen (Cx32 is a negative gater). Of course, this is not observed with Vj polarity reversal (**B**) because while the fast Vj-gates open at Cx32wt side (positive Vj side) they close at the tandem side (negative Vj side). From [77].

We first proposed the “cork” gating hypothesis based on the unusual behavior of certain Cx32 mutants heterotypically paired with Cx32 wild-type [4,37,77] (Figure 7 and Figure 8).

Since the behavior of these heterotypic channels is qualitatively the same, we will focus here just on channels made of Cx32tandems paired with Cx32 wild-type (tandem-32); a tandem is a dimer in which two Cx32 monomers are concatenated NT-to-CT (Figure 7A). Tandem-32 channels displayed a unique Ij-Vj behavior (Figure 7B,D). With tandem side negative, as Vj was increased stepwise from 20 to 120 mV initial and final Ij progressively decreased to very low values and the channels were sensitive to even the lowest Vj values (−20 mV). With tandem side positive, Ij gradually increased to high values and up to Vj = 80mV Ij monitored at the end of the pulse was higher than initial Ij (Figure 7D). This Vj behavior indicates that Vj positive at mutant gradually renders operational more channels. Significantly, this asymmetric behavior is absent following inhibition of CaM expression (Figure 7E) [37].

To test the idea that Vj positive at the tandem side renders operational a greater number of channels, the effect of applying repeated long 60 mV Vj pulses (tandem side positive) was tested (Figure 7C). The following Ij behaviors were observed: monophasic Ij rise (pulses 1–3); biphasic Ij time-course (pulses 4–9), displaying an initial gradual Ij rise followed by exponential Ij decay; and ultimately a fairly conventional Ij behavior (pulses 10–18). The application of conventional Vj protocols (tandem side positive), after the train of Vj pulses, resulted in an Ij behavior similar to that of homotypic Cx32 channels (Figure 7C; pulses 19–27). This clearly suggested that Vj positive at mutant side renders progressively operational numerous previously “dormant” (gated) channels.

To determine the time-course of the Gj changes in response to positive and negative Vj at the tandem side, tandem-32 channels were subjected to long, steady-state, Vj gradients [77] (Figure 8A,B). In oocytes initially clamped at Vm = −20 mV (Vj = 0), 40–80 mV steady-state Vj gradients (tandem side positive) slowly and exponentially increased Gj (τ = ~1 min) by as much as 400% (Figure 8A,B). In contrast, steady-state Vj gradients negative at the tandem side reduce Gj by over 85% (Figure 8A,B). Upon returning to Vj = 0 from Vj = 40 mV (tandem side positive), Gj suddenly increased before dropping (Figure 7A,B). This was caused by the reopening of fast Vj-gates at the Cx32 side (Cx32 is a negative gater). As predicted, this did not occur when Vj polarity was switched from positive to negative (Figure 8B), as in this case while fast Vj-gates open at the Cx32wt’s side (positive Vj side) fast Vj-gates close at the tandem side (negative Vj side). 

**Figure 9:**
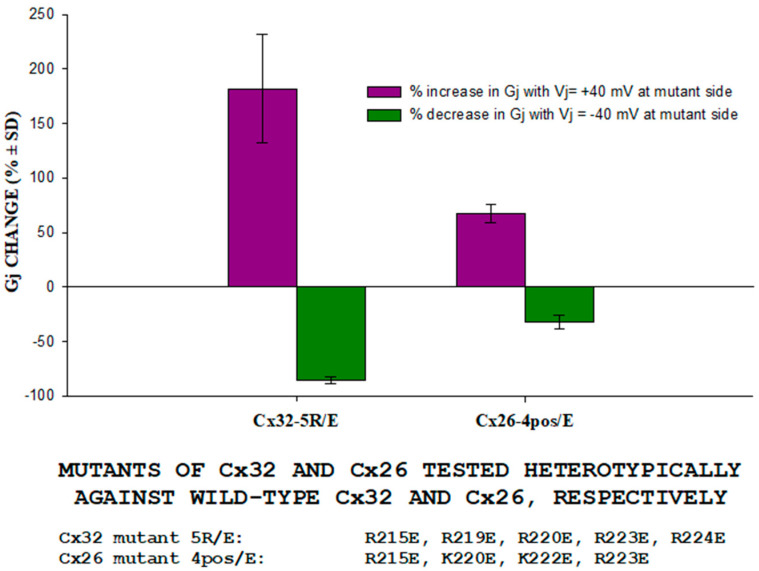
Percent Gj change in oocytes expressing Cx32-mutant (Cx32-5R/E) or Cx26-mutant (Cx26/4pos-E) heterotypic channels. Cx26-4pos/E channels behaved as Cx32-5R/E (or tandem-32) channels when subjected to Vj gradients, in spite of the fact that their fast Vj gates are activated by opposite Vj polarities (Cx32 is “negative gater”, Cx26 is “positive gater”). This confirms that the slow Gj change with Vj gradients is a gating behavior based on the activity of the chemical/slow gate, which is clearly distinct from that of the fast Vj gate1]. Adapted from [1].

The slow change in Gj is clearly consistent with the gating behavior of the chemical/slow gate, which is clearly different from the behavior of the fast Vj-gate. There are many reasons for this distinction [77], one is that in all connexins tested the chemical/slow gate closes at the negative Vj side, whereas the fast Vj-gate closes at the negative or positive Vj side depending on the connexin type. In fact, heterotypic channels (4pos/E-26) between Cx26 and a Cx26 mutant (4pos/E), in which four basic CT residues are mutated to acidic residues (E), behave qualitatively as heterotypic tandem-32 (or 5R/E-32) channels when subjected to Vj gradients (Figure 9) [53]. This is important because the fast Vj-gates of Cx26 and Cx32 are activated by opposite Vj polarities—Cx32 is a “negative gater” while Cx26 is a “positive gater” [78]. This obviously suggests that in Cx32 and Cx26 mutant channels this gating behavior results from the activity of the negatively charged chemical/slow gate (CaM’s N-lobe?). Significantly, the asymmetrical Vj behavior of heterotypic mutant-Cx32 channels disappeared with inhibition of CaM expression (Figure 7E) [37]. Based on these data and single-channel records (see in the following), we proposed the “CaM-Cork” gating model [4] (see in the following).

### 3.2. Chemical/Slow Gating Behavior at the Single-Channel Level

**Figure 10:**
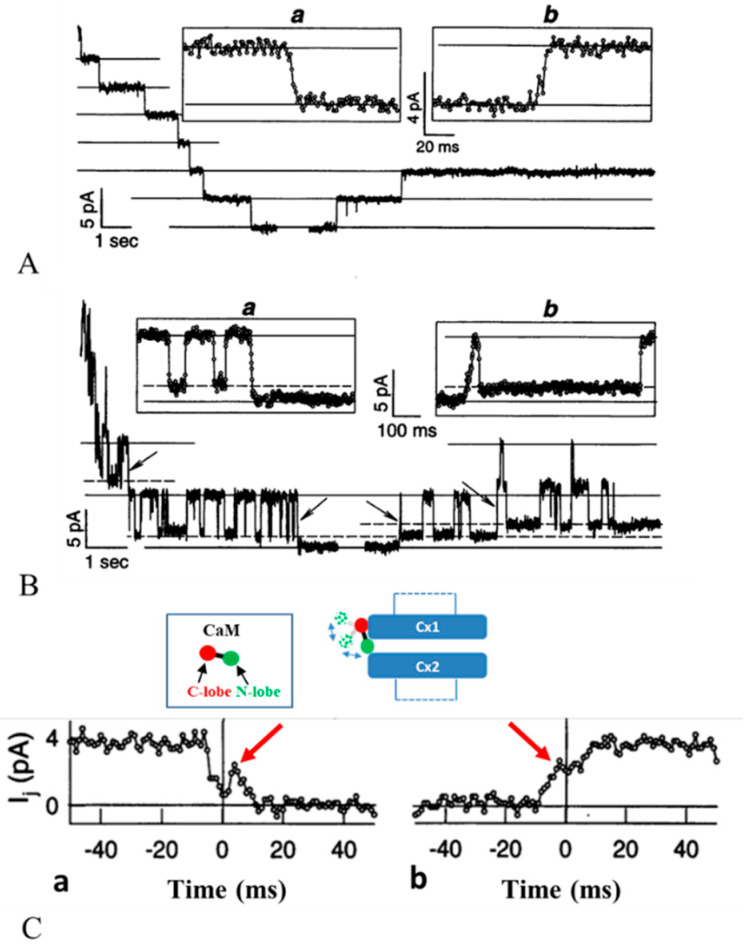
Effects of 100% CO_2_ on single-channel gating behavior in gap junctions of fibroblast pairs subjected to Vj 30 (**A**) or 55 (**B**) mV. Gating was monitored before total uncoupling ((**A**,**B**) left traces) and at the initial recoupling phase ((**A**,**B**) right traces). With Vj = 30 mV (**A**), each channel closes by a slow Ij transition from open, yj(main state), to closed state ((**A**) left trace and inset (**Aa**)) and reopens with a slow transition from closed state to yj(main state)((**A**) right trace and inset (**Ab**)). With Vj = 55 mV (**B**), the channels show two Ij transition: (1) between open and closed state (120 pS, ~10 ms; (**B**) left trace, arrows and inset (**Ba**)), and (2) between open and residual state (90 pS, 2 ms; (**B**) left trace and inset (**Ba**)). The channels reopen by a slow Ij transition to open state ((**B**) right trace, arrows and inset (**Bb**)), followed by fast flickering to residual state. Thus, the chemical gate closes slowly and completely, while the fast Vj gate closes fast and partially. Chemical gate transitions between open and fully closed states (**Ca**) and vice versa (**Cb**), often display fluctuations (**C**, red arrows), suggesting that the gate (CaM’s N-lobe?) may flicker in and out of the channel’s mouth before settling (**C**, inset). From [79].

The behavior of the chemical/slow gate was studied by a double whole-cell clamp (DWCC) at minimal Gj values in rat fibroblasts and Cx43-expressing HeLa cells during exposure to 100% CO_2_ [79]. We recorded junctional current (Ij), single-channel conductance (γj) and Ij-kinetics, during uncoupling and recoupling at three Vj gradients (Figure 10). Since the fast Vj-gate is only activated by Vj gradients >40–50 mV, by monitoring gating at three Vj gradients: Vj = 30 mV (fast Vj-gate open; Figure 10A), Vj = 55 mV (fast Vj-gate flickering; Figure 10B) or Vj = 70 mV (fast Vj-gate mostly closed), we were able to study in detail the individual behavior of the chemical/slow gate and the fast Vj gate.

With Vj = 30 mV, CO_2_ just caused slow Ij transitions from open to closed channel states (transition time = ~10 ms; chemical/slow gating activity; Figure 10A). With Vj = 55 mV, we monitored fast Ij flickering between open (γj main state) and residual (γj residual) states (transition time = ~2 ms; fast Vj gate activity) and slow Ij transitions between open and closed channel states (chemical/slow gate activity; Figure 10B). With Vj = 70 mV, aside from slow Ij transitions between open and closed channel states, CO_2_ caused slow transitions between residual and closed channel states (chemical/slow gate activity) [79].

During recoupling, the channels reopened, a slow transition (transition time = ~10 ms) from closed to open state (chemical/slow gate activity; Figure 10A,B), was followed by fast Ij flickering between open and residual state (fast Vj gate activity; Figure 10B). These data agree with earlier reports on insect cells [80] and mammalian cells expressing Cx40 [81]. 

Significantly, chemical/slow gating transitions between open and fully closed states and vice versa often display fluctuations (Figure 10C, red arrows). This suggests that the gate, likely to be CaM’s N-lobe, may flicker in and out of the channel’s mouth before settling.

Later studies provided further evidence for Vj sensitivity of the chemical/slow gate [37,53,77,82] and for a CaM role in its behavior [37,38]. These findings confirm evidence that the chemical/slow gate behaves like a slow Vj-sensitive gate. In the absence of uncouplers, chemical/slow gating activity is seldom observed. An exception are Cx45 channels, several of which are closed by the chemical/slow gate even at resting [Ca^2+^]_i_ and pH_i_ [38,74]. In addition to Cx45 channels, the chemical/slow gate is occasionally active in various channels made of connexin mutants (see in the following) and is activated in wild-type Cx32 channels by large Vj gradients [82] (see in the following).

### 3.3. The Chemical/Slow Gate of Cx32 Channels Closes with Vj Gradients

**Figure 11:**
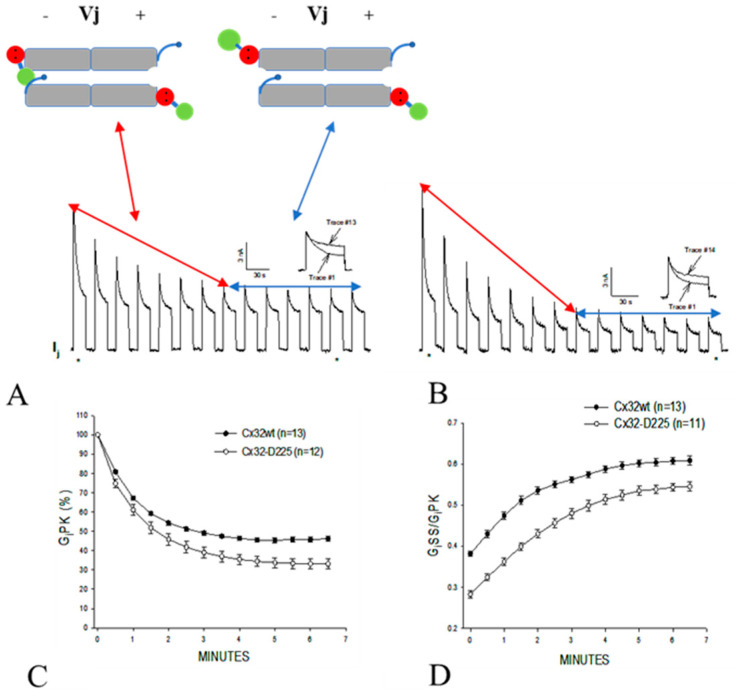
Slow Gj decay in oocytes expressing either Cx32 or its CT-truncated mutant (Cx32-D225) subjected to long −100 mV Vj pulses. (**A**,**B**) show junctional current (Ij) tracings recorded from Cx32wt and Cx32-D225, respectively. Note the gradual drop in Ij-Peak (IjPK) and, to a lesser extent, Ij-Steady-State (IjSS; A and B). GjPK drops exponentially by 50–60%, eventually reaching steady state (**C**). GjSS/GjPK increases by 60% and 93% in Cx32wt and Cx32-D225, respectively (**D**). The large drop in Ij during the first 8 pulses is likely to result from the closure of both chemical/slow gate and fast Vj gate ((**A**,**B**) double-headed red arrows and left drawing). The Ij behavior of the following 6 Vj pulses is likely to reflect the closure of only the fast Vj gate of the remaining open channels ((**A**,**B**) double-headed blue arrows and right drawing). From [82].

It has been generally thought that the chemical/slow gate is mostly inactive in the absence of uncouplers or connexin mutations. In contrast, in 2007 we reported that this gate can be activated by applying series of large Vj gradients (Figure 11) [82]. In channels expressing Cx32 wild-type (Figure 11A) or the mutant Cx32-D225 (CT-truncated at res. 225; Figure 11B), the application of series of –100 mV Vj pulses induces peak (IjPK) and steady-state (IjSS) Ij to gradually drop (Figure 11A,B) [82]. Gj steady state (GjSS) drops less dramatically that Gj peak (GjPK), so that the ratio GjSS/GjPK progressively increases (Figure 11D). Gj peak drops exponentially with the first 6–7 Vj pulses by 50–60%, eventually reaching a steady state (Figure 11C). Most likely, while the first 6–7 pulses activate both chemical/slow gate and fast Vj gate, the following pulses only activate the fast Vj gate of the remaining open channels (Figure 11A,B). Gj, monitored during recovery by applying small Vj pulses, slowly recovers, often achieving values greater that initial ones. Greater effects were obtained with the mutant Cx32-D225 [82] (Figure 11B–D), indicating that this degree of CT deletion does not eliminate the efficiency of the chemical/slow gate, but rather renders it more Vj-sensitive.

**Figure 12:**
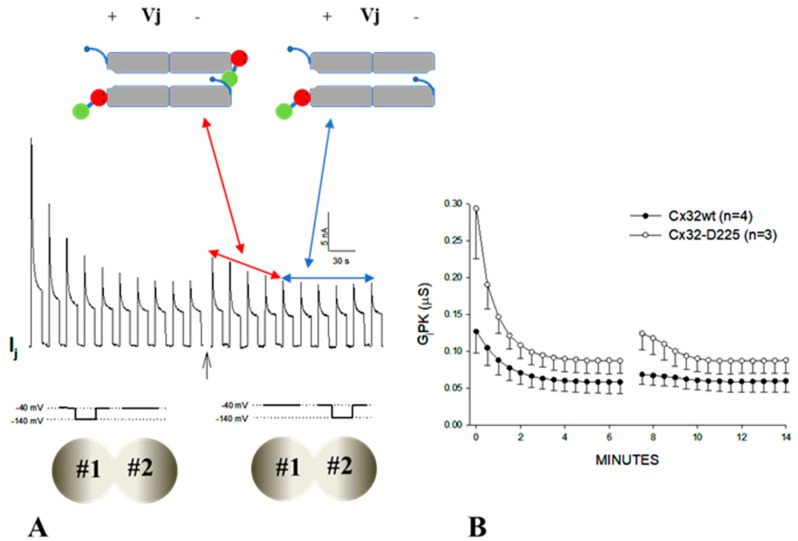
Effect of Vj polarity-reversal on junctional conductance (Gj) in oocyte pairs expressing either Cx32 subjected to Vj pulses of -100 mV. The Ij and GjPK records ((**A**,**B**) respectively) show that in the first few pulses of the second series peak Ij (IjPK; **A**, arrow) and consequentially GjPK (**B**) are substantially greater than that of the last pulse of the first series. This indicates that positive Vj is more effective in opening hemichannels than negative Vj in closing them. Significantly, this phenomenon is more pronounced with Cx32-D225 channels (**B**). From [82].

With Vj polarity reversal, GjPK and GjSS increase slightly before dropping, as compared to the last few pulses of the previous series, (Figure 12A,B). Since both fast Vj gate and chemical/slow gate of Cx32 channels close at the negative side of Vj with Vj polarity reversal, previously the closed hemichannel gates, now at positive Vj side, start opening, while those at the negative Vj side start closing. If opening and closing kinetics were identical, GjPK and GjSS would not change from the last pulse of the series and the first pulse of the new series. However, since GjPK and GjSS are higher following Vj polarity reversal it is likely that Vj positive is more efficient and faster at opening hemichannel than Vj negative at closing them. Based on our hypothesis that the chemical/slow gate is the N-lobe of CaM, these data would suggest that the N-lobe’s displacement by positive Vj from the channels’ mouth is more rapid than its insertion into the mouth by negative Vj. Since this phenomenon is more pronounced with Cx32-D225 channels (Figure 12A,B) it is possible that the absence of the CT domain facilitates the displacement of the CaM’s N-lobe away from the channel’s mouth. The CT deletion may also facilitate channel plugging by the chemical gate, because the drop in IjPK and GjPK is much greater with Cx32-D225 than Cx32 wild-type channels (Figure 11A–C and Figure 12B). 

These data clearly indicate that the gate responsible for the exponential Gj drop is the chemical/slow gate, and not the fast Vj-gate. The presence of chemical/slow gating activity in Cx32′s wild-type channels indicates that this gate (CaM’s N-lobe?) can be made operational by large Vj gradients even in the absence of uncouplers and/or mutations. In fact, these data [82] confirm earlier evidence of Vj-induced slow-gating events to zero channel conductance in Cx32-expressing cells [83]. The behaviors previously described confirm the direct role of CaM in chemical/slow gating (CaM-Cork mechanism) because they are all strongly reduced by the inhibition of CaM expression [1,37,38]. 

## 4. Calmodulin Cork Model—One Molecule, Two Mechanisms

**Figure 13:**
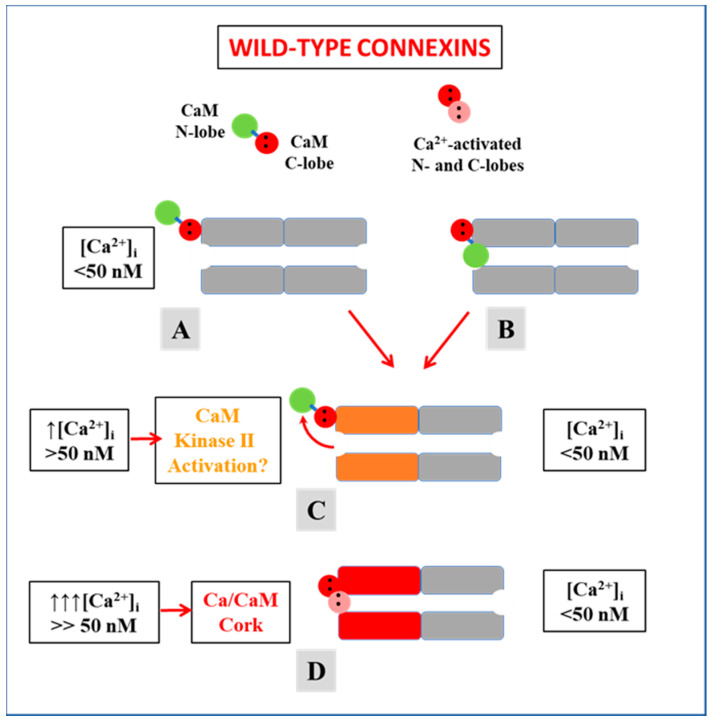
Schematic representation of Ca-CaM-Cork gating. Our hypothesis is that under normal conditions, while most channels are open (**A**) some are closed (**B**) by the CaM-Cork mechanism (**B**). With a small [Ca^2+^]_i_ rise above resting values, CaMKII becomes activated, possibly resulting in the opening of CaM-Cork gated channels (**C**; orange connexins). With greater [Ca^2+^]_i_ rise the Ca^2+^-activated CaM’s N-lobe interacts hydrophobically and electrostatically with the connexin’s gating site and plugs the channel’s pore (**D**; red connexins), probably also causing conformational changes in connexins (**D**).

Based on data described previously, we have proposed two mechanisms for the CaM-driven gating model. In one, “Ca-CaM-Cork”, gating is initiated by Ca^2+^-induced activation of the CaM’s N-lobe (Figure 13D). In the other, “CaM-Cork”, gating occurs without a [Ca^2+^]_i_ rise above resting values. Ca-CaM-Cork gating is only reversed by a return of [Ca^2+^]_i_ to resting values, while CaM-Cork gating is reversed by Vj positive at the gated side.

### 4.1. Ca-CaM-Cork Gating

**Figure 14:**
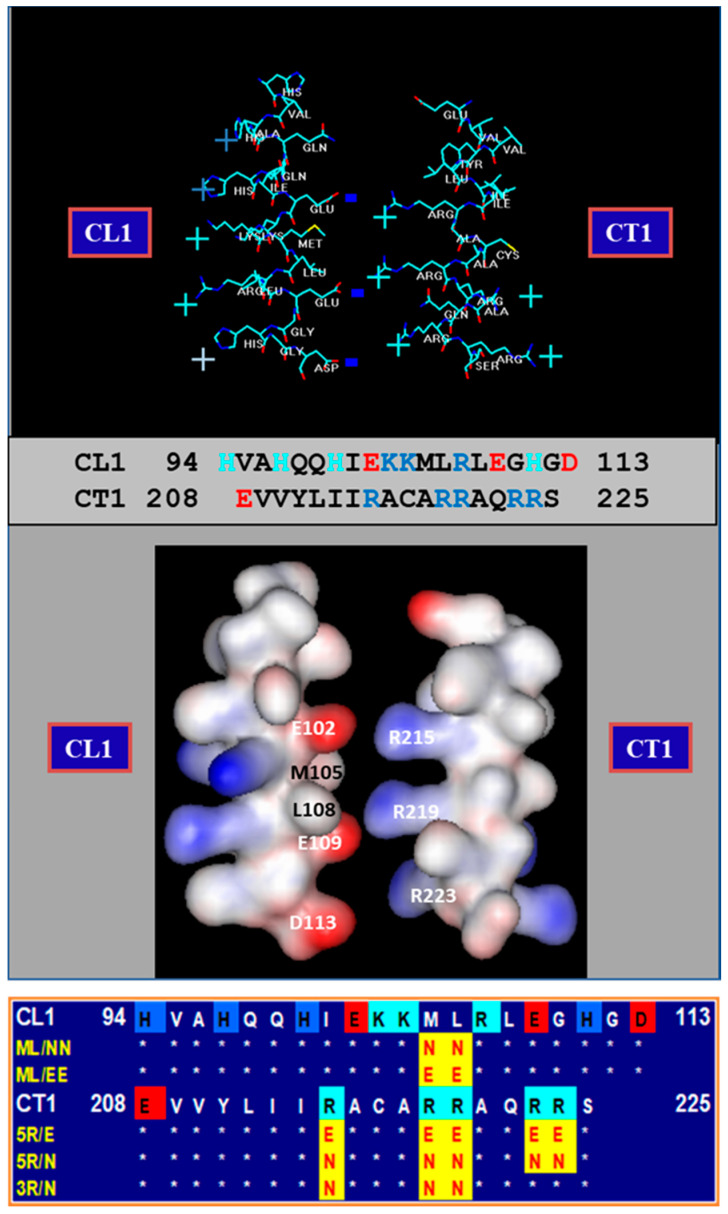
Cx32′s CL1 (first half of CL) and CT1 (initial domain of CT) domains in α-helical conformation. Our hypothesis is that in coupled conditions CL1 and CT1 interact electrostatically (negative CL1, positive CT1) and hydrophobically. CL1 and CT1 mutation (bottom panel) are believed to prevent the interaction allowing the negatively charged CaM’s N-lobe to access the positively charged channel’s mouth and plugging it by interaction with it electrostatically (CaM-Cork model). We suggest that with an increase in [Ca^2+^]_i_ the activated CaM’s N-lobe accesses the gating site and plugs the channel’s mouth by breaking the CL1-CT1 interaction (Ca-CaM-Cork model).

There is evidence that in most coupled cells, especially in those expressing Cx45, a number of channels are in closed state even at resting [Ca^2+^]_i_ [38,74] (Figure 13A,B) [38,84]. We believe that some channels in closed state at rest (Figure 13B) might be gated by the CaM-Cork mechanism (see in the following). Our hypothesis is that with a small [Ca^2+^]_i_ rise above resting values the CaMKII cascade is likely to be activated, resulting in the opening of CaM-Cork gated channels (Figure 13C)—see initial Gj rise in Figure 2 and Figure 3. In contrast, greater [Ca^2+^]_i_ rise would activate the CaM’s N-lobe, enabling it to bind to a CaM’s connexin site and plug the channel’s mouth (Figure 13D) [1,4,53]. At resting [Ca^2+^]_i_, CaM is believed to be linked to each connexin by the C-lobe, probably at the CL2 site (Figure 5) [55,56], while the N-lobe in most channels is likely to be free. In Cx32 channels, the CaM’s lobe may be unable to access the channel’s mouth by the postulated CL1-CT1 interaction (Figure 14) [1,64].

The C-lobe’s Ca^2+^-affinity constant is greater than that of the N-lobe by almost one order of magnitude [41,85]. Thus, it is likely that the N-lobe only interacts with the connexin’s gating site (CL2 or NT) with [Ca^2+^]_i_ rises above resting levels. This model agrees with evidence for independent functions of N- and C-lobes in the interaction with Cx32 [59].

With a [Ca^2+^]_i_ rise, one possibility is that in all of the six CaMs anchored to the connexon the N-lobes are activated, bind to the NT or CL2 sites of the same connexin and change connexins conformation. The conformational change would allow one of the six N-lobes to access the channel’s mouth and plug the pore by binding to the NT or CL2 site of the opposite connexin hydrophobically and electrostatically. Another possibility is that while all of the N-lobes are activated one wins the competition (first comes first served), binds to a site (NT or CL2) of the opposite connexin and plugs the pore by interacting with the site hydrophobically and electrostatically.

### 4.2. CaM-Cork Gating

**Figure 15:**
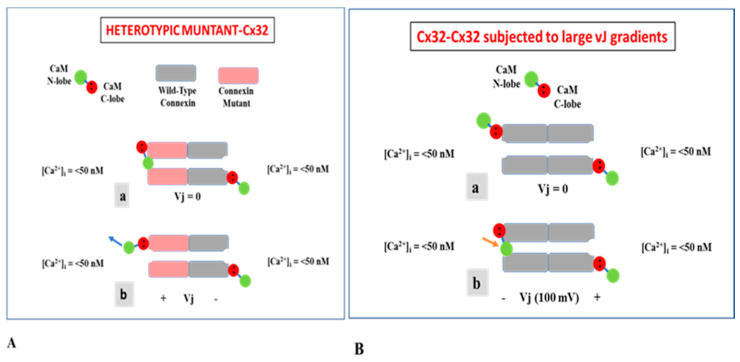
Schematic representation of CaM-Cork gating. CaM is anchored to connexins by its C-lobe at resting [Ca^2+^]_i_ (**A**,**B**). Certain connexin mutations enable the negatively charged CaM’s N-lobe to access the channel’s pore and plug it by interacting electrostatically with the positively charged channel’s mouth even at resting [Ca^2+^]_i_ (**Aa**); the N-lobe can be moved out of the pore with Vj gradients positive at the mutant side (**Ab**). In wild-type connexins (Cx32-CX32), with the application of large Vj gradients the N-lobe can be reversibly forced to plug the channel’s mouth at the negative side of Vj at resting [Ca^2+^]_i_ (**Bb**).

**Figure 16:**
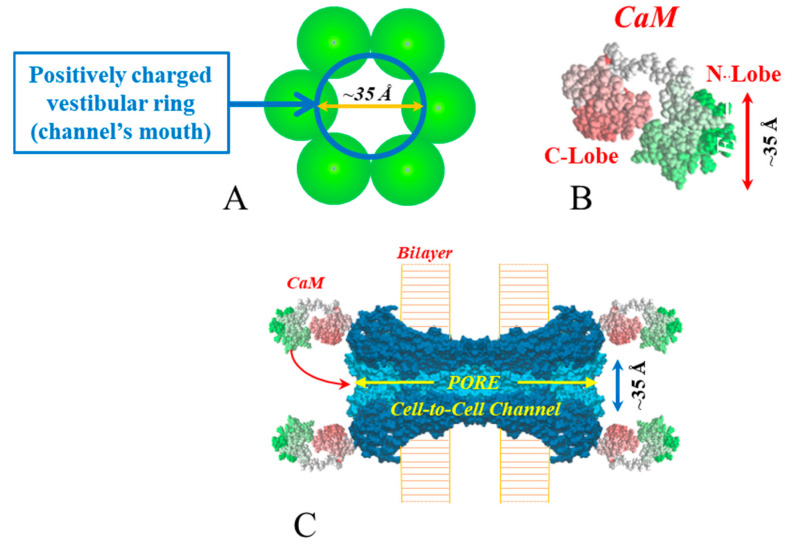
Both the positively charged channel’s mouth (**A**,**C**) and the negatively charged CaM lobes (**B**) are ~25 × 35 Å in size. Therefore, a CaM lobe could interact and fit well within the positively charged connexon’s mouth (vestibule) (**A**,**C**). In (**C**) the channel is split lengthwise so that the pore diameter (light blue area) is seen throughout the entire channel’s length. Both the CaM and connexon images (**B**,**C**) were provided by Drs. Francesco Zonta and Mario Bortolozzi (VIMM, University of Padua, Italy).

The CaM-Cork mechanism is demonstrated by the behavior of heterotypic mutant-Cx32 channels (Figure 15A) [37,77], homotypic Cx45 channels and Cx32 channels subjected to large Vj gradients negative at the gating site (Figure 15B) [82]. Our hypothesis is that at the mutant side of mutant-Cx32 and mutant-Cx26 channels (see previously) a CaM’s N-lobe can access the channel’s mouth even at resting [Ca^2+^]_i,_ (Figure 15Aa)—perhaps, the mutations make the channel’s mouth unprotected. The N-lobe would interact only electrostatically with the channel’s mouth, such that Vj positive at the mutant hemichannel’s side could release it from the mouth (Figure 15Ab) [1,37,77]. Significantly, both the negatively charged CaM lobes and the positively charged channel’s mouth are ~35 Å in size (Figure 16A–C).

As previously pointed out, the behavior of CaM-Cork gating is a manifestation of the chemical-slow gate (CaM’s N-lobe?) rather than that of the fast Vj-gate because Vj positive at mutant side opens channels of both “negative” (Cx32) and positive (Cx26) fast Vj-gaters (Figure 9) [1,4]. If the fast Vj-gates were involved, Vj positive at the mutant side would close, not open mutant-Cx26 channels (4pos/E-26).

Cytoplasmic domains of connexins have a high basic/acidic amino acid ratio. In Cx32, for example, neglecting most of CT whose deletion by 84% has no effect on gating sensitivity [63,64], and assigning values of one for R, K, D and E and 1/2 for H, one counts 18 basic and six acidic residues per connexin—108 and 36, respectively, per connexon. In view of the effectiveness of short-range electric fields, the Vj-sensitive slow-gating behavior of mutant Cx32 channels would be expected to manifest itself only if the gating CaM lobe were near the channel’s mouth.

Why would the channel’s mouth be accessible to the CaM’s N-lobe only in Cx32 mutants? Perhaps, in Cx32 and other connexins, the channel’s mouth is not very accessible due to the postulated CL1-CT1 electrostatic/hydrophobic interaction (Figure 14) [1,37,64,77,86]. The potential absence of this interaction in mutant channels lacking CT1′s positive charges (Figure 14; 5R/N, 5R/T and 3R/N), in mutant channels in which charges are converted to negative (Figure 14; 5R/E) [64,77,86], and in mutant channels in which the hydrophobic CL1′s amino acids M105 and L106 are mutated to N or E (Figure 13; ML/NN, ML/EE, ML/NN + 3R/N) [37], would be expected to enable the N-lobe to access the channel’s mouth. In tandem-Cx32 channels, the NT-CT linkage in tandem might prevent the proposed CL1-CT1 interaction [37,77]. 

Based on evidence for the effect on Gj of large Vj gradients applied to Cx32 channels (Figure 10 and Figure 11) [82], it seems possible that the gating element (CaM’s N-lobe?) could be forced to plug the mouth of the hemichannel subjected to negative Vj in the absence of connexin mutations or Ca^2+^-activation. Thus, the hemichannel’s mouth might not be completely inaccessible [82]. 

In some channels made of wild-type connexins, Vj gradients may not be needed for forcing the gating particle (CaM’s N-lobe?) into the channel’s mouth. In fact, in Cx45-expressing cells, for example, many channels are closed by the chemical/slow gate at Vj = 0 and resting [Ca^2+^]_i_ [38,74]. In these cases, the closed channels are likely to be gated by the CaM-Cork mechanism and are likely to be opened by small increases in [Ca^2+^]_i_ possibly via activation of the CaMKII cascade. Perhaps, the postulated CL1-CT1 interaction is weaker in Cx45 channels. Since inhibition of CaM expression inhibits the chemical gating of Cx45 channels [38], the gating element is likely to be a CaM lobe electrostatically bound to the channel’s pore.

## 5. Conclusion

Evidence that gap junction mediated cell communication is finely regulated by nanomolar [Ca^2+^]_i_ via the direct action of Ca^2+^-CaM indicates that gap junction channel gating is not just a safety mechanism for protecting cells from damaged/dead neighbors (healing-over). Rather, it is also a mechanism designed to finely modulate cell–cell exchange of small molecules.

We have proposed a two-facet CaM-mediated gating mechanism: Ca-CaM-Cork and CaM-Cork. In summary:

1. At resting [Ca^2+^]_i_, (<50nM) some channels are spontaneously closed by the CaM-Cork gating mechanism.
2. With moderate [Ca^2+^]_i_ rise (50–100 nM, the CaMKII cascade may be activated causing channels closed by the CaM-Cork mechanism to open.
3. With greater [Ca^2+^]_i_ rise (>100 nM), the channels start closing by the Ca-CaM-Cork mechanism. CaM lobe channel mouth plugging is likely to include connexin conformational changes.
4. CaM-Cork gated channels could be reopened by Vj positive at gated side, but since they would close at the negative side no Gj change would occur. This is not the case with heterotypic channels between wild-type connexins paired with more gating-sensitive mutants.
5. Most Ca-CaM-Cork gated channels reopen with a drop in [Ca^2+^]_i_ to resting values (<50 nM). However, with prolonged exposure to high [Ca^2+^]_i_, channel gating may not be reversible.

Many questions still need to be answered in terms of molecular details, such as: Is CaM anchored to the NT or the CL2 domain? Is CaM anchored to connexins by the C-lobe or the N-lobe? Is the gating lobe the N-lobe or the C-lobe? Does the gating lobe bind to the CL2 or the NT CaM binding site? Are all of the CaMs anchored to a connexon Ca^2+^-activated? If so, how many lobes gate the channel? Does CaM activation cause connexin conformational changes?

## Figures and Tables

**Table 1:** CaM binding to CL2.

Connexins	kD (With Ca^2+^)	kD (Without Ca^2+^)
Cx32	40 ± 4 nM	280 ± 10 nM
Cx35	31 ± 2 nM	2,67 ± 0.09 µM
Cx45	75 ± 4 nM	78 ± 1 nM
Cx57	60 ± 6 nM	32 ± 14 nM
